# Supplementary material for: A Highly Sensitive Next-Generation Sequencing-Based Genotyping Platform for EGFR Mutations in Plasma from Non-Small Cell Lung Cancer Patients
Source: Cancers (Basel). 2020 Nov 30;12(12):3579. doi: 10.3390/cancers12123579 (PMC7760633; doi:10.3390/cancers12123579)
Supplement: Supplementary file 1 [file cancers-12-03579-s001.pdf]

cancers

Supplementary Materials

A Highly Sensitive Next-Generation Sequencing-Based Genotyping Platform for *EGFR* Mutations in Plasma from Non-Small Cell Lung Cancer Patients

Jung-Young Shin, Jeong-Oh Kim, Mi-Ran Lee, Seo Ree Kim, Kyongmin Sarah Beck and Jin Hyoung Kang

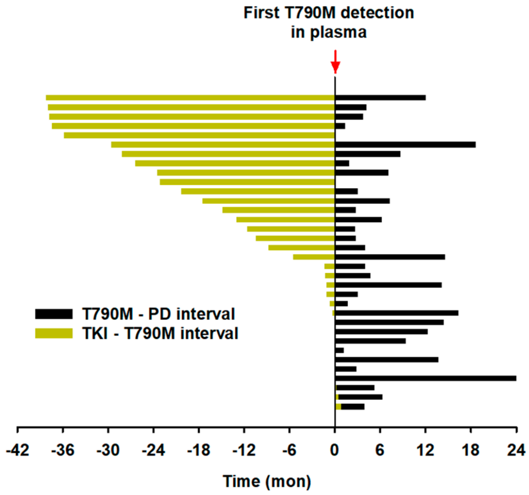

Figure S1. Timeline plots showing intervals between EGFR-TKI initiation and first T790M detection (yellow color), and intervals between first T790M detection and disease progression (black color) in patients (n = 34) who developed drug resistance to first-line EGFR-TKI treatments.

Table S1. Diagnostic performance of Sel-Cap and NGS tumor biopsies for two *EGFR* activating mutations in NSCLC patients (n = 61), with paired PNAclamp tumor biopsy as the reference.

		Ex19del						L858R					
		Sel-Cap			NGS			Sel-Cap			NGS		
		Mutant	Wild	Total	Mutant	Wild	Total	Mutant	Wild	Total	Mutant	Wild	Total
PNAclamp	Mutant	20	0	20	18	2	20	16	1	17	16	1	17
	Wild	0	41	41	0	41	41	0	44	44	0	44	44
	Total	20	41	61	18	43	61	16	45	61	16	45	61
Sensitivity	(95% CI)	100% (84% - 100%)			90% (70% - 97%)			94% (73% - 99%)			94% (73% - 99%)		
Specificity	(95% CI)	100% (91% - 100%)			100% (91% - 100%)			100% (92% - 100%)			100% (92% - 100%)		
Accuracy	(95% CI)	100% (94% - 100%)			97% (89% - 99%)			98% (91% - 100%)			98% (91% - 100%)		
Kappa	(95% CI)	1.00 (1.00 - 1.00)			0.92 (0.82 - 1.03)			0.96 (0.88 - 1.04)			0.96 (0.88 - 1.04)		

Table S2. Clinical characteristics of patients included in the retrospective longitudinal monitoring study (n = 34).

No.	T90M Detection Group	Sex	Age	Stage at TKI	Previous Chemo	EGFR-TKI	Companion Mutation	T790M Detected at # Sampling	Interval TKI - T790M (mon)	Interval T790M - PD (mon)	PFS
1	Early	F	63	IV	N	afatinib	L858R	1st	-0.8	3.9	3.1
2	Early	M	77	IV	N	erlotinib	Ex19del	1st	-0.5	6.3	5.9
3	Early	M	61	IV	N	afatinib	Ex19del	1st	-0.2	5.2	4.9
4	Early	F	67	IV	unknown	afatinib	L858R	1st	-0.1	24.2	24.1
5	Early	F	81	IIIA	N	gefitinib	L858R	1st	-0.1	2.9	2.8
6	Early	F	75	IV	N	gefitinib	Ex19del	1st	0.0	13.7	13.7
7	Early	M	54	IV	Y	afatinib	Ex19del	1st	0.0	1.2	1.2
8	Early	M	50	IV	N	gefitinib	Ex19del	1st	0.0	9.4	9.4
9	Early	M	80	IV	unknown	erlotinib	Ex19del	1st	0.0	12.3	12.3
10	Early	M	65	IIIB	N	afatinib	L858R	1st	0.0	14.4	14.4
11	Early	F	64	IV	N	afatinib	Ex19del	1st	0.3	16.3	16.6
12	Early	F	77	IIIA	unknown	erlotinib	L858R	1st	0.7	1.7	2.4
13	Early	F	57	IV	N	erlotinib	L858R	1st	1.1	3.0	4.1
14	Early	M	59	IV	N	afatinib	Ex19del	1st	1.1	14.1	15.2

15	Early	F	72	IV	N	gefitinib	Ex19del	1st	1.3	4.7	6.0
16	Early	M	56	IIB	Y	afatinib	None	1st	1.4	4.0	5.5
**No.**	**T90M Detection Group**	**Sex**	**Age**	**Stage at TKI**	**Previous Chemo**	**EGFR-TKI**	**Companion Mutation**	**T790M Detected at # Sampling**	**Interval TKI - T790M (mon)**	**Interval T790M - PD (mon)**	**PFS**
17	Late	F	39	IV	N	afatinib	Ex19del	2nd	5.5	14.6	20.1
18	Late	F	43	IV	N	gefitinib	L858R	2nd	8.8	4.0	12.8
19	Late	F	73	IV	N	gefitinib	Ex19del	2nd	10.5	2.8	13.3
20	Late	M	64	IV	N	afatinib	Ex19del	4th	11.6	2.7	14.3
21	Late	F	68	IV	Y	erlotinib	None	4th	13.0	6.2	19.2
22	Late	M	48	IV	N	gefitinib	Ex19del	3rd	14.9	2.8	17.7
23	Late	F	51	IIB	N	erlotinib	L858R	5th	17.5	7.3	24.8
24	Late	M	75	IV	N	erlotinib	Ex19del	3rd	20.3	3.0	23.3
25	Late	M	61	IV	N	gefitinib	L858R	5th	23.2	0.0	23.2
26	Late	M	66	IV	N	gefitinib	Ex19del	4th	23.5	7.1	30.5
27	Late	F	53	IV	N	erlotinib	Ex19del	3rd	26.4	1.9	28.3
28	Late	F	69	IV	Y	erlotinib	None	6th	28.2	8.7	36.8
29	Late	M	71	IIIA	Y	erlotinib	Ex19del	4th	29.6	18.6	48.2
30	Late	M	50	IIIB	Y	erlotinib	L858R	4th	35.8	0.0	35.8
31	Late	F	61	IIIA	N	erlotinib	Ex19del	4th	37.4	1.4	38.8
32	Late	F	69	IIIA	N	erlotinib	None	4th	37.8	3.7	41.5
33	Late	F	46	IIB	N	gefitinib	Ex19del	5th	38.0	4.2	42.2
34	Late	F	74	IV	N	erlotinib	L858R	4th	38.2	12.0	50.2

© 2020 by the authors. Licensee MDPI, Basel, Switzerland. This article is an open access article distributed under the terms and conditions of the Creative Commons Attribution (CC BY) license (http://creativecommons.org/licenses/by/4.0/).
